# Magnetization Characteristics of Oriented Single-Crystalline NiFe-Cu Nanocubes Precipitated in a Cu-Rich Matrix

**DOI:** 10.3390/molecules25143282

**Published:** 2020-07-19

**Authors:** Shota Kobayashi, Tsuyoshi Yamaminami, Hibiki Sakakura, Mahoto Takeda, Tsutomu Yamada, Hiroshi Sakuma, Suko Bagus Trisnanto, Satoshi Ota, Yasushi Takemura

**Affiliations:** 1 Department of Electrical and Computer Engineering, Yokohama National University, Yokohama 240-8501, Japan; kobayashi-shota-pb@ynu.jp (S.K.); yamaminami-tsuyoshi-jx@ynu.jp (T.Y.); yamada@ynu.ac.jp (T.Y.); suko-trisnanto-zt@ynu.ac.jp (S.B.T.); 2 Department of Materials Engineering, Yokohama National University, Yokohama 240-8501, Japan; pluto.0609@gmail.com (H.S.); takeda@em.elec.mie-u.ac.jp (M.T.); 3 Department of Fundamental Engineering, School of Engineering, Utsunomiya University, Utsunomiya 321-8585, Japan; hsakuma@cc.utsunomiya-u.ac.jp; 4 Department of Electrical and Electronic Engineering, Shizuoka University, Hamamatsu 432-8561, Japan; ota.s@shizuoka.ac.jp

**Keywords:** magnetic nanostructure, single-crystalline nanocubes, magnetocrystalline anisotropy, dynamic magnetization process, specific loss power

## Abstract

In this study, we evaluated the magnetization properties of a magnetic alloy with single-crystalline cubic nanostructures, in order to clarify its magnetocrystalline anisotropy. Upon applying a specific annealing treatment to the CuNiFe base material, the precipitated magnetic particles grew into cubic granules, resulting in the formation of nanometric cubic single crystals of magnetic CuNiFe in a nonmagnetic Cu-rich matrix. The cubic nanostructures of CuNiFe were oriented along their crystallographic axis, in the <100> direction of the face-centered-cubic structure. We evaluated the static magnetization properties of the sample, which originated primarily from the CuNiFe nanocubes precipitated in the Cu-rich matrix, under an applied DC magnetic field. The magnetocrystalline anisotropy was readily observed in the magnetization curves. The <111> axis of the CuNiFe was observed to be the easy axis of magnetization. We also investigated the dynamic magnetization properties of the sample under an AC magnetic field. By subtracting the magnetic signal induced by the eddy current from the magnetization curves of the sample, we could obtain the intrinsic AC magnetization properties of the CuNiFe nanocubes.

## 1. Introduction

Hyperthermia is a therapeutic procedure used for cancer treatment, which is used to elevate the body temperature above 42.5 °C. The magnetic hyperthermia treatment using magnetic nanoparticles [1,2] relies on the generation of heat from magnetic nanoparticles under the applied AC magnetic field. Much attention has been paid to improving the specific loss power (SLP), which is also referred to as the specific absorption rate of magnetic nanoparticles, and to clarifying the relationship between the SLP and the shape magnetic anisotropy of magnetic nanoparticles [3,4,5,6]. Magnetocrystalline anisotropy is another case of magnetic anisotropy that originates from the internal energy, depending on the direction in the crystal lattice of a magnetic material. Magnetocrystalline anisotropy is not observed in conventional magnetic nanoparticle samples, such as fluids or powders, in which the crystal axes are not aligned. In the case of magnetic nanoparticles dispersed in a liquid, the rotation degree and the relaxation time of the easy axis of each particle are influenced by the magnetic anisotropy [7,8]. The magnetic anisotropy is increased by dipole interaction in chainlike structures [9,10], and decreased in multicore structures [11,12] of magnetic nanoparticles, respectively. The effects of the anisotropy constant and primary particle volume associated with the anisotropy energy on the magnetization dynamics were individually evaluated by numerical simulation [13]. However, in order to develop magnetic nanoparticles for hyperthermia and other biomedical applications, it is necessary to analyze the magnetocrystalline anisotropy of nanoparticles. In studies related to magnetic recording, the magnetocrystalline anisotropy of an L1_0_-FePt alloy has attracted attention, as it is a magnetic material with higher magnetic anisotropy constants [14]. While most experimental and theoretical studies on magnetocrystalline anisotropy have been conducted on bulk and with thin film materials [15,16,17,18], very few studies have been reported on magnetic nanoparticles. Although the effect of the uniaxial magnetic anisotropy of magnetic nanoparticles on the SLP has been studied theoretically [19], it is important to evaluate the magnetocrystalline anisotropy of magnetic nanoparticles according to experimentally evaluated characteristics.

In this study, we evaluated the magnetic properties of single-crystalline magnetic nanostructures of CuNiFe nanocubes oriented along their crystallographic axis, <100> of the face-centered-cubic (FCC) crystal structure. The magnetization properties were evaluated under a magnetic field applied along the <100>, <110>, and <111> axes of the nanocubes. Both the static and the dynamic magnetization properties were evaluated by applying DC and AC magnetic fields, respectively. As regards the AC magnetization properties, an eddy current was induced in the conductive sample used in this study. We evaluated the AC magnetization of the sample by applying a DC bias magnetic field simultaneously, at an intensity that was sufficient to saturate the magnetization of the sample. Only the magnetic signal from the eddy current induced by the applied AC magnetic field was observed under the DC bias field. After eliminating this magnetic signal, we could derive the intrinsic AC magnetization of the sample and its magnetic anisotropy.

## 2. Results

### 2.1. Materials

In this study, ingots of a Cu_75_Ni_20_Fe_5_ (composition in at.%) alloy were used as the base material. This sample was subjected to solution treatment at 1323 K for 360 min in vacuum capsules, and then quenched in ice-cold water. Thereafter, the samples contained in capsules were subjected to an aging treatment: they were heated at 873 K for 1000 min in an electric furnace. Details of the sample preparation can be found in [20,21] and the articles cited therein. The sample for measurement was cut into a cube with side lengths of 2 mm and a mass of 76.1 mg.

**Figure 1:**
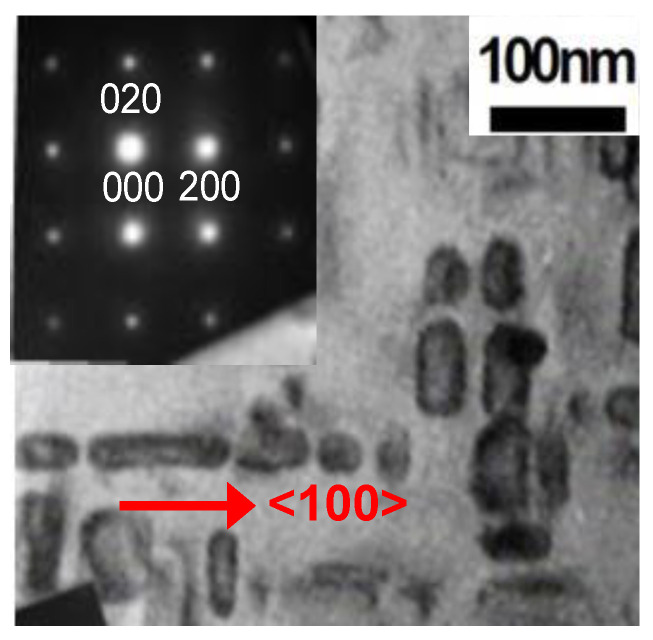
Bright-field transmission electron micrograph and the selected area electron diffraction pattern of the Cu_75_Ni_20_Fe_5_ sample.

**Figure 2:**
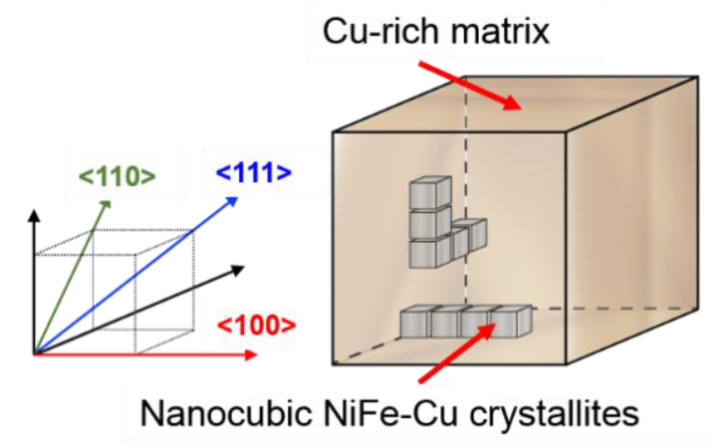
Schematics of the structure and crystal orientations of the Cu_75_Ni_20_Fe_5_ sample.

Figure 1 shows the microstructure of the sample processed from the Cu_75_Ni_20_Fe_5_ alloy, as observed by bright-field transmission electron microscopy (TEM). Cubic nanostructures were precipitated in the sample. The nanometer-sized small particles, which were randomly distributed within the base material, appeared during the early stage of the aging treatment. They grew into spherical precipitates with sizes ≤10 nm, and finally, single cubic crystalline granules that were oriented along the <100> crystallographic axis of the matrix [20] were obtained. Each side of the precipitated nanocubes was found to be approximately 30 nm in length. The nanocubes were aggregated and formed rectangular blocks that were aligned in the <100> direction of the matrix. Figure 1, which shows the electron diffraction pattern of the selected area of the sample, indicates that both the matrix and the nanocubes crystallized with FCC structures, and that their crystal orientations were identical [21]. The electron back-scattering diffraction inverse pole figure map obtained revealed that the sample was single crystalline, and its crystal direction covered an area of approximately 2 mm × 2 mm [20]. Figure 2 shows the schematics of the structure and the crystal orientations of the sample. The atomic compositions of the matrix and the precipitated nanocubes determined by energy-dispersive X-ray spectroscopic analysis, using a scanning transmission electron microscope, are shown in Table 1. The ratio of the copper-rich matrix to the nanocubes was calculated to be 87.5:12.5 by thermodynamic analysis. By calculating the electron energy band using first principles with the Korringa–Kohn–Rostoker coherent potential approximation (KKR-CPA) method, the density of states and magnetic moments per atom *μ_B_* were derived. The calculated *μ_B_* values are listed in Table 1. From these analyses, we concluded that the precipitated nanocubes of Cu_21.2_Ni_51.4_Fe_27.4_ (hereafter denoted as NiFe-Cu nanocubes) are ferromagnetic, while the Cu-rich matrix is substantially nonmagnetic [22]. We also confirmed the nonmagnetic nature of the as-quenched CuNi alloy from its magnetization curve obtained at 5 K with a magnetic field up to 2390 kA/m, using a SQUID magnetometer [23]. According to the phase diagram and Curie temperature variation of CuNiFe alloys reported in earlier publications [24], Cu_21.2_Ni_51.4_Fe_27.4_ and Cu_80.9_Ni_16.6_Fe_2.5_ alloys are stable in the crystal structure of FCC, and their Curie temperatures are approximately 700 K and considerably lower than room temperature, respectively. These previously reported characteristics are in accordance with the experimentally determined characteristics. Therefore, the magnetism of the sample studied in this work stemmed from the NiFe-Cu nanocubes. Here, we report the magnetization characteristics of the NiFe-Cu nanocubes and their magnetocrystalline anisotropy.

### 2.2. Measurements

DC (static) magnetization measurement and AC (dynamic) magnetization measurement of the NiFe-Cu nanocubes were performed. The direction of the applied magnetic field corresponded with each of the crystallographic axes, <100>, <110>, and <111>. The DC magnetization curves were obtained using a vibrating sample magnetometer at room temperature. The maximal intensities of the applied DC magnetic field, *H*_DC_, were 1200 kA/m for the major loop, and 4, 8, 16, 32, 64, and 128 kA/m for the minor loop. The AC magnetization curves were obtained using a 20-turn pickup coil that was wound around the sample. The pickup coil had an inner diameter of 3.8 mm and a length of 2.0 mm, which enabled it to entirely cover the sample. The maximal intensity of the applied AC magnetic field, *H*_AC_, was 4 kA/m, and its frequency was varied in the range of 1–100 kHz. When an AC magnetic field is applied to the sample, an eddy current is generated according to the Faraday’s law, both in the conductive NiFe-Cu nanocubes and the Cu-rich matrix. As a result, the pickup coil detects the magnetic field generated by the eddy current as well as the magnetization of the NiFe-Cu nanocubes in the AC magnetization measurement. In order to evaluate the AC magnetization property of the NiFe-Cu nanocubes, the AC magnetization was measured with and without an applied DC bias magnetic field, *H*_B_, of 1200 kA/m. The magnetization of the NiFe-Cu nanocubes was saturated by this DC bias field, resulting in a lack of induced AC magnetization in the sample. The details of this method of eliminating the effect of the eddy current in the sample are described in Section 3.2.

## 3. Discussion

### 3.1. DC Magnetization Curves

**Figure 3:**
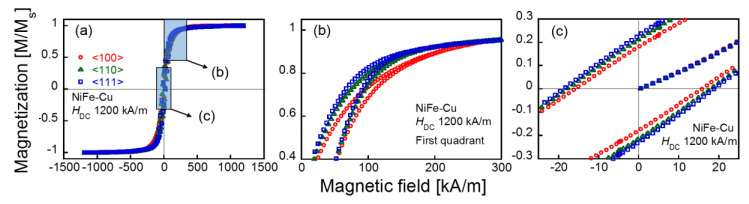
DC magnetization curves (major loop) of the NiFe-Cu nanocubes recorded with the maximal DC magnetic field, *H*_DC,_ of 1200 kA/m (**a**), and their enlarged views (**b**,**c**). ((**c**) includes the initial magnetization curves traced from the virgin state of the sample).

**Figure 4:**
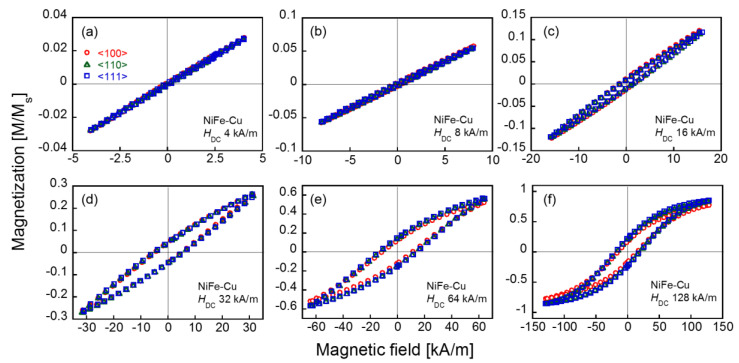
DC magnetization curves (minor loops) of the NiFe-Cu nanocubes recorded with the applied field intensities *H*_DC_ of 4 kA/m (**a**), 8 kA/m (**b**), 16 kA/m (**c**), 32 kA/m (**d**), 64 kA/m (**e**), and 128 kA/m (**f**).

**Figure 5:**
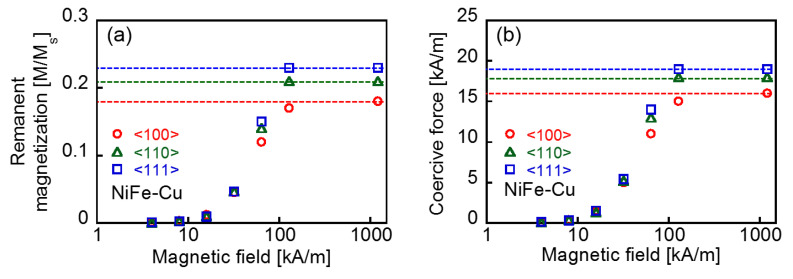
Remanent magnetization (**a**) and coercive force (**b**) of the DC minor loops obtained under different applied magnetic field intensities, *H*_DC_, of the NiFe-Cu nanocubes. The dotted lines indicate each value obtained from the DC major loops (*H*_DC_ = 1200 kA/m) shown in Figure 3.

As the magnetization of the Cu-rich matrix was negligible compared to that of the NiFe-Cu nanocubes [22], the measured magnetic property of the Cu_75_Ni_20_Fe_5_ sample was considered to have originated primarily from the NiFe-Cu nanocubes. Figure 3 shows the magnetization curve (major loop) obtained under the maximal DC magnetic field, *H*_DC_, of 1200 kA/m applied along each crystallographic direction of the NiFe-Cu nanocubes. The values of the measured magnetization are normalized by the saturation magnetization of the sample, and indicated as *M*/*M*_S_. The saturation magnetization, *M*_S_, was found to be 116 kA/m. The TEM image shown in Figure 1 suggests that the NiFe-Cu nanocubes have shape anisotropy in their magnetization, and predicts that the <100> direction is an easy axis along the longer axis of the rectangular aggregates. Nevertheless, Figure 3b shows a larger magnetization under the applied magnetic field along the <111> direction than that obtained by applying the magnetic field along the <100> direction. The remanent magnetization for the <111> direction was slightly larger than that for the <100> direction, as shown in Figure 3c. Regardless of the size of the nanocubes, which was in the order of 10 nm, the sample did not exhibit superparamagnetism. Figure 4 shows the DC minor loops of the sample obtained under various *H*_DC_. The remanent magnetization and coercive force derived from these minor loops are shown in Figure 5. Both the remanent magnetization and coercive force were independent of the crystallographic direction of the applied DC field for *H*_DC_ <64 kA/m. They increased with increasing *H*_DC_, and almost saturated at *H*_DC_ = 128 kA/m. The saturated values of the remanent magnetization and coercive force obtained from the DC major loops (*H*_DC_ = 1200 kA/m) are also plotted as dotted lines in the figures. The remanent magnetization and coercive force for the <111> direction were larger than those for the <100> direction. The magnetization curves shown in Figure 3, along with these analyses, suggest the magnetocrystalline anisotropy of the NiFe-Cu nanocubes and the ease of magnetization along the <111> direction. The NiFe-Cu nanocubes have the FCC crystal structure and their composition is rich in Ni, as shown in Table 1. Accordingly, the easy magnetization axis along the <111> direction in the NiFe-Cu nanocubes is appropriately similar to that of Ni with the FCC crystal structure [25,26].

Energy density of the magnetocrystalline anisotropy of cubic crystals, *E*_A_ is given by [26,27,28]

(1) $$E_{A}=K_{0}+K_{1}\left(\alpha_{1}^{2}\alpha_{2}^{2}+\alpha_{2}^{2}\alpha_{3}^{2}+\alpha_{3}^{2}\alpha_{1}^{1}\right)+K_{2}\left(\alpha_{1}^{2}\alpha_{2}^{2}\alpha_{3}^{2}\right)+\cdots ,$$

where *K*_0_, *K*_1_, and *K*_2_ are the magnetocrystalline anisotropy constants and α_1_, α_2_, and α_3_ are the direction cosine of the magnetization to each axis of *hkl* coordinates of the cubic crystal, respectively. The energy density required to magnetize a sample in the crystallographic axis <*hkl*>, $E_{hkl}$ is

(2) $$E_{hkl}=\mu_{0}\int_{0}^{M}H_{hkl}dM_{hkl},$$

where, *μ*_0_ is the permeability of free space. $E_{100}=K_{0}$, $E_{110}=K_{0}+\frac{K_{1}}{4}$, and $E_{111}=K_{0}+\frac{K_{1}}{3}+\frac{K_{2}}{27}$. From these equations as well as the measured initial magnetization curves of the sample traced from its virgin state, the magnetocrystalline anisotropy constants are calculated to be *K*_1_ = −1.3 × 10^3^ J/m^3^ and *K*_2_ = −3.9 × 10^3^ J/m^3^. Both *K*_1_ and *K*_2_ are negative, similar to the magnetocrystalline anisotropy constants of Ni with an FCC crystal structure, which exhibits easy magnetization along the <111> direction. The atomic ratio of Ni/Fe of the sample is 65/35, which is in the range of that of the NiFe alloy, a permalloy. The *K*_1_ of the permalloy varies depending on the order phase or disorder phase, the composition, and the heat treatment. Nevertheless, it is normally small, of the order of $\left|K_{1}\right|=$1 kJ/m^3^ [15].

### 3.2. AC Magnetization Curves

**Figure 6:**
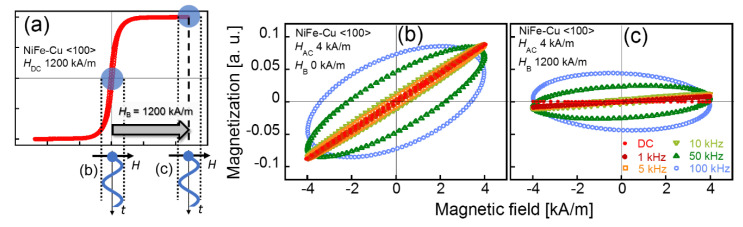
DC magnetization curve along the <100> crystallographic axis of the NiFe-Cu nanocubes, and a schematic of the applied AC field and DC bias field for AC magnetization measurement (**a**), AC magnetization curves of the sample obtained without the DC bias field (*H*_B_ = 0) (**b**), and with a DC bias field (*H*_B_ = 1200 kA/m) (**c**).

The evaluation of the dynamic magnetization properties of magnetic nanostructures is essential for developing practical applications as well as for understanding their physics. Figure 6a shows the DC magnetization curve of the sample obtained with *H*_DC_ = 1200 kA/m along the <100> direction. It also shows a schematic indicating the method of AC magnetization measurement with and without the DC bias field, *H*_B_ = 1200 kA/m. Figure 6b,c show the AC magnetization curves of the sample under the bias fields of *H*_B_ = 0 and 1200 kA/m, respectively. The highest frequency of *H*_AC_ used for the AC magnetization measurement in this study was 100 kHz. The skin depth of Cu at 100 kHz was 0.21 mm, which suggests that *H*_AC_ was not substantially applied to the entire volume (depth) of the sample. However, the measured values of magnetization did not decrease under the applied field frequency, up to 100 kHz, as shown in Figure 6b. The magnetization shown in Figure 6b is the result of the alternating magnetization reversal of the ferromagnetic NiFe-Cu nanocubes as well as the alternating magnetic field induced by the eddy current, whereas the magnetization shown in Figure 6c only originated from the alternating magnetic signal induced by the eddy current. This is because the magnetization was saturated by applying a bias field, *H*_B,_ of 1200 kA/m, and no modulation of magnetization was facilitated by the applied AC field, *H*_AC,_ of 4 kA/m. The magnetic signal induced by the eddy current was almost zero at 1 kHz, and it increased with an increase in the applied field frequency. The effect of the eddy current induced by the applied AC magnetic field was negligible at a low frequency, but was enhanced at a higher frequency.

**Figure 7:**
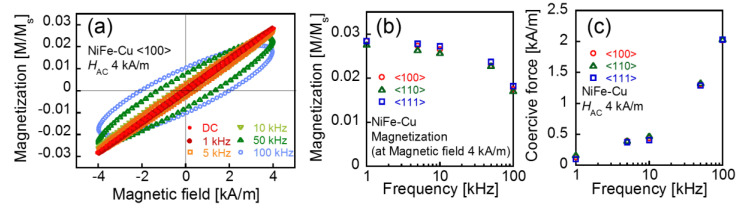
Intrinsic AC magnetization curves of the NiFe-Cu nanocubes for the <100> crystallographic axis (**a**), frequency characteristics of maximum magnetization (**b**), and coercive force (**c**).

Figure 7 shows the intrinsic AC magnetization of the NiFe-Cu nanocubes that arose only from the magnetization reversal in response to the applied AC magnetic field. The magnetization curves were calculated by subtracting the waveform of the magnetization measured under *H*_B_ = 1200 kA/m from that measured under *H*_B_ = 0. The subtraction of magnetization curves is a validated method in extracting specific magnetization dynamics from superposed complex dynamics [29]. The AC magnetization curves and their dependence on frequency shown in the figure are quite similar to those of the magnetic iron oxide nanoparticles [30,31]. The magnetization curves obtained under the applied DC field or AC field at the low frequency of 1 kHz exhibit approximately no remanent magnetization. The dependences of the maximum magnetization at *H*_AC_ = 4 kA/m, and of coercive force on the applied field frequency, are shown in Figure 7b,c. The magnetization decreased while the coercive force increased with increasing frequency. This was caused by a delay in the response of magnetization reversal against the change in the applied field [32].

The magnetization of magnetic nanoparticles was reversed by the Néel relaxation and Brownian relaxation processes. These are the result of the rotation of magnetization in each particle and the rotation of the particle itself, respectively [33,34,35]. As the NiFe-Cu nanocubes were dispersed in a solid Cu-rich matrix, the magnetization reversal of the nanocubes was accompanied only by the Néel relaxation process. The Néel relaxation time, *τ_N_*, of a magnetic nanostructure exhibiting a single magnetic domain is expressed as

(3) $$\tau_{N}=\tau_{0}\exp\left(\frac{K_{a}V_{c}}{k_{B}T}\right),$$

where *τ*_0_ (=1.0 × 10^‒9^ s), *K*_a_, *V*_c_, *k*_B_, and *T* (=300 K) denote the attempt time, anisotropy constant, volume of the sample, Boltzmann constant, and temperature, respectively. *K*_a_ is determined to be 580 J/m^3^ by $\left|E_{111}-E_{100}\right|=\left|\frac{K_{1}}{3}+\frac{K_{2}}{27}\right|$, where <111> and <100> are the easy and hard axes of the sample, respectively. Here, we employed the volume of the NiFe-Cu nanocubes as *V*_c_, which is reasonable for Equation (3), assuming the magnetization reversal of the single domain structure. The Néel relaxation time was calculated to be 43 ns, which corresponds to the frequency of 3.7 MHz. The magnetization response is delayed with respect to the applied AC magnetic field even at 50 and 100 kHz, as shown in Figure 7. Nevertheless, this is also commonly observed in the AC magnetization curves of magnetic nanoparticles [36]. Because of its exponential dependence on the volume and practical distribution of the size of the NiFe-Cu nanocubes, the Néel relaxation time of the nanocubes presumably varies over a wide range of the order of 10 kHz to 100 MHz. The frequency dependence of the obtained magnetization curves clearly indicates the typical feature of a magnetic nanostructure of a single domain.

As shown in Figure 7b,c, both the maximum magnetization and coercive force are independent of the direction of the applied AC field along the crystallographic axes of <100>, <111>, and <110>. As shown in Figure 5, the magnetocrystalline anisotropy between these axes is observed under applied magnetic fields larger than 50 kA/m, which cannot be practically achieved with an AC magnetic field of 10 kHz or higher. In general, a larger applied field intensity than the anisotropy energy is necessary to observe the anisotropic properties. Therefore, the magnetocrystalline anisotropy was not observed during the dynamic magnetization process in this measurement under *H*_AC_ = 4 kA/m. The anisotropic features in a dynamic magnetization process are observed when the magnetic field larger than the anisotropy energy is applied to the samples [11,37,38,39].

The SLP is derived by

(4) $$SLP=\frac{f\mu_{0}\oint^{\;}HdM}{\rho },$$

where *f* and *ρ* are the frequency of the applied AC magnetic field and the mass density of the sample, respectively. The SLP of the NiFe-Cu nanocube under the applied field of *H*_AC_ = 4 kA/m at 100 kHz is calculated to be 1.6 W/g from the magnetization curve along the <100> direction, shown in Figure 7a, which does not depend on the crystallographic direction of the applied field. This value of SLP is comparable with that of Resovist^®^ (SLP = 3.2 W/g), calculated for the same AC field condition [4]. Resovist^®^ is a contrast agent that is clinically used in magnetic resonance imaging, which also exhibits a high SLP value for hyperthermia. We concluded that the NiFe-Cu nanocubes are an attractive material that can be used as a heating agent, and that a high temperature increase can be expected from them under the applied AC field intensity and frequency sufficient for hyperthermia.

## 4. Conclusions

In this study, the magnetization of the single-crystalline NiFe-Cu nanocubes was studied in order to evaluate the magnetocrystalline anisotropy of the magnetic nanostructures. The NiFe-Cu nanocubes were precipitated in the matrix of a Cu-rich alloy by applying a specific annealing treatment. Both the nanocubes and matrix were found to be single-crystalline with an FCC structure, and they were aligned along their identical crystallographic axes. The static magnetization curves of the NiFe-Cu exhibited anisotropic features depending on the crystallographic axis, which suggests the easy axis of magnetization to be the <111> axis. Anisotropic constants of *K*_1_ = −1.3 × 10^3^ J/m^3^ and *K*_2_ = −3.9 × 10^3^ J/m^3^ were obtained from the magnetization curves. In the dynamic magnetization curve, the applied AC magnetic field reversed the magnetization and simultaneously induced the eddy current in the conductive sample. By recording the dynamic magnetization curves under the DC bias magnetic field, which saturated the magnetization of the NiFe-Cu nanocube, we could quantify the magnetic signal arising from the eddy current. The intrinsic properties of the dynamic magnetization were derived by subtracting the contribution of the eddy current from the measured magnetization. A delay in the magnetization reversal in response to the applied AC field was observed in the intrinsic hysteresis loops. Owing to the limited intensity of the applied AC magnetic field, the magnetocrystalline anisotropy of the sample was not observed in the dynamic magnetization measurement. The NiFe-Cu nanocubes exhibit a high SLP of 1.6 W/g under the applied AC field of 4 kA/m at 100 kHz, which is an attractive feature for hyperthermia applications.

## Figures and Tables

**Table 1:** Atomic compositions determined by energy-dispersive X-ray spectroscopy analysis using a scanning transmission electron microscope, magnetic moments per atom calculated from first principles analysis of the matrix, and nanocubic precipitates prepared from the base material of Cu_75_Ni_20_Fe_5_ alloy.

Base Material: Cu_75_Ni_20_Fe_5_	Cu	Ni	Fe	*μ_B_*
Matrix	80.9	16.6	2.5	0.04
Nanocubic precipitates	21.2	51.4	27.4	1.00
